# Intestinal parasitic infections in relation to HIV/AIDS status, diarrhea and CD4 T-cell count

**DOI:** 10.1186/1471-2334-9-155

**Published:** 2009-09-18

**Authors:** Shimelis Assefa, Berhanu Erko, Girmay Medhin, Zelalem Assefa, Techalew Shimelis

**Affiliations:** 1 Department of Medical Laboratory Science, Gondar University, PO Box 196 Gondar, Ethiopia; 2 Aklilu Lemma Institute of Pathobiology, Addis Ababa University, PO Box 1176, Addis Ababa, Ethiopia; 3 Department of Surgery, Addis Ababa University, PO Box 16466, Addis Ababa, Ethiopia; 4 Department of Medical Laboratory Science, Hawassa University, PO Box 1560, Hawassa, Ethiopia

## Abstract

**Background:**

HIV infection has been modifying both the epidemiology and outcome of parasitic infections. Hence, this study was undertaken to determine the prevalence of intestinal parasitic infection among people with and without HIV infection and its association with diarrhea and CD4 T-cell count.

**Methods:**

A cross-sectional study was conducted at Hawassa Teaching and Referral Hospital focusing on HIV positive individuals, who gave blood for CD4 T-cell count at their first enrolment and clients tested HIV negative from November, 2008 to March, 2009. Data on socio-demographic factors and diarrhea status were obtained by interviewing 378 consecutive participants (214 HIV positive and 164 HIV negative). Stool samples were collected from all study subjects and examined for parasites using direct, formol-ether and modified acid fast stain techniques.

**Results:**

The prevalence of any intestinal parasitic infection was significantly higher among HIV positive participants. Specifically, rate of infection with *Cryptosporidium*, *I. belli*, and *S. stercoralis *were higher, particularly in those with CD4 count less than 200 cells/μL. Diarrhea was more frequent also at the same lower CD4 T-cell counts.

**Conclusion:**

Immunodeficiency increased the risk of having opportunistic parasites and diarrhea. Therefore; raising patient immune status and screening at least for those treatable parasites is important.

## Background

Intestinal parasites are endemic in many regions of the world where Human Immunodeficiency Virus/Acquired Immunodeficiency syndrome (HIV/AIDS) is also prevalent. Sub-Saharan Africa is among the regions where intestinal parasitic infections are entrenched [1] and the largest burden of AIDS cases exist [2]. The same factors including poverty and malnutrition could promote transmission of both infections in the region, and attempt to improve the underlying conditions may revert the situations.

Studies investigated the existence of interaction between HIV and parasitic infections in co-infected individuals. Parasitic infections particularly helminths cause chronic immune activation [3,4], in addition to skewing the immune response toward T helper-2 immune responses [5]. Though proving evidences are insufficient, such immune modulation was shown to increase host susceptibility; thereby, promoting HIV infection and disease progression [6-8]. Thus, chronic immune activation was suggested as one factor that adversely influences epidemics of HIV/AIDS in Africa [9].

On the other hand, HIV infection has increased significance of parasitic infection. More importantly, with emergence of AIDS, the epidemiology as well as outcome of diseases caused by opportunistic parasites was significantly modified [10,11]. But, the effect of HIV on some other parasites is not clearly understood. Overall, either backed by HIV or independently, intestinal parasitic infections have continued to be major cause of morbidity and mortality in humans [12].

Ethiopia is among the sub-Saharan countries with overlapping high rate of HIV and parasitic infections. However, there have been few studies to ascertain whether epidemiology of intestinal parasites take different picture as population with HIV/AIDS is growing. The present study is, therefore, aimed to determine the prevalence and pattern of intestinal parasites in people with and without HIV infection and also their association with diarrhea and immune status.

## Methods

This cross-sectional study was conducted at Hawassa Teaching and Referral Hospital, situated in Hawassa City, southern Ethiopia from November, 2008 to March 2009. The hospital uses two approaches for HIV/AIDS counseling and testing: client-initiated to serve people seeking to know their HIV status and provider-initiated to enable health care provider offer specific medical services. People who test HIV-positive at the hospital and referred from other health institutions are attached to the antiretroviral therapy (ART) clinic for clinical and laboratory investigations to monitor their disease status. Immunological assessment using CD4+ T-cell count at enrollment and every three months follow-up visits helps to identify those who are eligible for ART.

The study population consisted of HIV-positive individuals, who gave blood for CD4 T-cell count at their first enrolment to monitor disease status at the ART clinic during the study period and clients tested HIV-negative at the hospital in the same period. Patients receiving anti-parasitic treatment were excluded from the study. In total, 378 consecutive clients (214 HIV positive and 164 HIV negative) were included in the present study.

All study subjects were interviewed on sociodemographic factors and asked about current symptom and duration of diarrhea. Diarrhea was defined as three or more watery or loose stools in 24-hour period. Persistence of diarrhea for more than two weeks period was considered as chronic; otherwise, it was acute diarrhea. On the same day of interview and CD4+ T-cell measurement, a single stool sample was collected from each participant and examined for intestinal parasites. Stool specimen were processed using direct technique (saline and iodine mounts) to identify trophozoite and cyst of protozoan parasites and using formol-ether concentration technique to detect eggs and larva of helminths. Modified acid fast stain was used to detect oocysts of *Cryptosporidium *species and *Isospora belli *[13].

Data entry and analysis was performed using SPSS Version-15 software. Different characteristics of study participants were described using mean, range and percentage as appropriate. Statistical significance of differences in proportions was evaluated by Pearson's Chi-square test. Fisher Exact test was used in place of Pearson's Chi-square when more than 20% of cells in contingency tables have expected count less than 5. Bivariate and multivariate logistic regression analyses were used to assess the crude and adjusted effects of HIV status and other pre-specified correlates on parasitic infection. Diarrhea as an ordered categorical outcome variable, we used proportional odds model to evaluate the independent effect of HIV and parasitic infection on diarrhea. Odds ratio was used to measure the strength of association between outcome and its correlates. A given statistical test was reported significant whenever it resulted in a p-value < 0.05.

The study was approved by the Ethics Committees of Aklilu Lemma Institute of Pathobiology, Addis Ababa University, and College of Health Sciences, Hawassa University. Participation was fully voluntary and informed written consent was obtained from each study subject. Physicians managed those participants positive for intestinal parasites.

## Results

A total of 378 individuals were screened for intestinal parasites during the study period. Majority of the participants were urban dwellers (80.7%) and females (57.4%). Thirty five subjects (9.3%) were less than 20-year old, 288 (76.2%) were in the age range 20 - 40-years and 55 (14.6%) were above 40-year old.

Of the total study subjects, participants with HIV infection were 56.6%. The mean age of HIV infected participants was 30.2 years (range 17-67 years; SD 8.8) compared with mean age of 27.2 years (range 14-71 years; SD 9.6) in HIV non-infected clients. The male to female ratio was 0.5:1 in HIV positive and 1.2:1 in HIV negative individuals.

During microscopic examination of stool samples fourteen types of parasite's genera were identified and 55.0% (208/378) of the study subjects were positive for at least one intestinal parasite. The most frequently detected parasites were *E. histolytica/dispar *(26.5%)and *A. lumbricodes *(12.4%) (Table 1). Infected individuals were found to harbor one and up to five types of parasites. Multiple infections (polyparasitism) were observed in 27.2% (103/378) of the total examined participants and in 49.5% (103/208) of those with parasitic infection.

**Table 1:** Distribution of different intestinal parasites among HIV positive and negative subjects, Hawassa Referral Hospital, Ethiopia, 2008

Type of parasite	HIV positive (N = 214) Number (%)	HIV-negative (N = 164) Number (%)	Total (N = 378) Number (%)	P-value
***Helminths ***				
*A. lumbricoides*	26(12.2)	21(12.8)	47(12.4)	0.85
*T. trichiura*	8(3.7)	13(7.9)	21(5.6)	0.08
*Hookworm *species	7(3.3)	13(7.9)	20(5.3)	0.05
*E. vermicularis*	2(0.9)	1(0.6)	3(0.8)	1.00*
*S. stercoralis*	27(12.6)	1(0.6)	28(7.4)	0.000
*Taenia species*	3(1.4)	5(3.1)	8(2.1)	0.30*
*H. nana*	4(1.9)	1(0.6)	5(1.3)	0.39*
*S. mansoni*	4(1.9)	4(2.4)	8(2.1)	0.73*
***Protozoa***				
*E. histolytica/dispar*	53(24.8)	47(28.7)	100(26.5)	0.40
*G. lamblia*	24(11.2)	12(7.3)	36(9.5)	0.20
*I. belli*	26(12.2)	0	26(6.9)	-
*Cryptosporidium *species	43(20.1)	0	43(11.4)	-
*E. coli*	4(1.9)	3(1.8)	7(1.9)	1.00*
*T. hominis*	2(0.9)	0	2(0.5)	-

* P value from Fisher's exact test

In bivariate analysis, the prevalence of parasitic infection was associated with gender and occupational status. After adjustment for significantly associated variables, farmers (odds ratio (OR) = 4.7; 95% CI 1.5 to 15.0) and merchants (OR = 3.0; 95% CI 1.2 to 7.5) had a higher risk of parasitic infection than civil servants. But, the association of gender and parasitic infection was non significant (OR = 1.4; 95% CI 0.9 to 2.3).

Among HIV positive subjects, 59.8% (128/214) were infected with one or more intestinal parasites compared with 48.8% (80/164) of HIV negatives (p = 0.033). This difference was still statistically significant in multivariate analysis (OR = 1.9; 95% CI 1.2 to 3.1). *Cryptosporidium *species and *I. belli *occurred exclusively among HIV positive individuals. Moreover, almost all cases (except one case) of *S. stercoralis *infection occurred among HIV positive participants. The association of hookworm infection with HIV status was marginally non-significant (p = 0.05).

Although single infection was not associated with HIV status, higher rate of mixed infection occurred among HIV positives than HIV negative individuals (OR = 2.0; 95% CI 1.2 to 3.6) (Table 2).

**Table 2:** Single and mixed infections among HIV positive and negative subjects, Hawassa Referral Hospital, Ethiopia, 2008

HIV status	Number of parasite species per individual				
	
	One	Two	Three	Four	Five
Positive (N = 214)	56(26.2)	46(21.5)	20(9.3)	5(2.3)	1(0.5)
Negative (N = 164)	49(29.9)	23(14.0)	6(3.7)	1(0.6)	1(0.5)
p-value*	0.25	0.04	0.03	0.19	1.00

*At any comparison the denominator is 214 for HIV positive group and 164 for HIV negative group.

In bivariate analysis, the association of diarrhea status was non-significant with HIV infection (p = 0.32) or parasitic infection (p = 0.05). Regardless of their parasitic infection status, HIV positive subjects reported increased rate of chronic diarrhea (Table 3). In a logistic regression model that adjusted for the effect of parasitic infection, HIV positive participants were 2.59 times more likely to have chronic diarrhea relative to HIV negative clients. In the same model, parasitic infection did not bring excess risk of chronic diarrhea. We also checked for interaction of HIV and parasitic infection in terms of their effect on chronic diarrhea and that interaction was not statistically significant (OR = 0.37, 95% CI: 0.11 to 1.18). This implies that the effect of HIV and parasitic infection was independent of each other in terms of effect they might have in exposing a person to experience chronic diarrhea.

**Table 3:** The occurrence of diarrhea among the study participants stratified by HIV and parasitic infection, Hawassa Referral Hospital, Ethiopia, 2008

Type of diarrhea	HIV positive		HIV Negative		Total		p-value
		
	Parasite positive	Parasite negative	Parasite positive	Parasite negative	HIV positive	HIV negative	
Acute	28(21.9%)	15(17.4%)	30(37.5%)	17(20.2%)	43(20.1%)	47(28.7%)	0.035
Chronic	29(22.7%)	25(29.1%)	12(15.0%)	7(8.3%)	54(25.2%)	19(11.6%)	0.001
No diarrhea	71(55.5%)	46(53.5%)	38(47.5%)	60(71.4%)	117(54.7%)	98(59.8%)	0.188
Total	128(59.8%)	86(40.2%)	80(48.8%)	84(51.2%)	214 (56.6%)	164(43.3%)	

**Table 4:** Distribution of diarrhea in different categories of CD4 T-cell counts, Hawassa Referral Hospital, Ethiopia, 2008

CD4 T-cell count/μL	Status of diarrhea			
	
	Total examined (%)	Diarrhea present (%)	Crude odds ratio (95% Confidence interval)	p-value
< 200	61 (28.5)	36 (59.0)	2.7 (1.24-5.91)	0.012
200-349	58 (27.1)	23(39.7)	1.24 (0.56-2.72)	0.597
350-499	46 (21.5)	21(45.7)	1.58 (0.69-3.61)	0.277
≥ 500	49 (22.9)	17(34.7)	1	

Total	214 (56.6%)	97 (45.3)		

Acute diarrhea was reported in higher rate among HIV negative subjects (p = 0.035). Of course, diarrhea is not expected to take an acute course among most HIV infected subjects who already have chronic diarrhea. Anyway, 37.5% (30/80) of HIV negative subjects with parasitic infection had reported to have acute diarrhea. This rate was significantly higher compared to HIV negatives with no parasitic infection and HIV positives with or without parasitic infection (p < 0.05). Similarly, HIV positive individuals with or without parasitic infections had reported higher rate of chronic diarrhea compared to HIV negative individuals with no parasitic infections (p < 0.05). In terms of causing any type of diarrhea (acute or chronic), HIV and parasite infection had significant interaction (OR = 0.33; 95% CI: 0.14, 0.78). Thus, the effect either of them should be interpreted in light of the infection status of the other.

In a proportional odds model where the comparison group was participants with no either infections (i.e. negative for HIV and parasitic infection), those with HIV and parasite co-infection had reported to have higher risk of any type of diarrhea or chronic diarrhea (OR = 2.18; 95% CI: 1.23, 3.88). Similarly, infection with HIV alone (OR = 2.60; 95% CI: 1.39 to 4.85) or parasite alone (OR = 2.40; 95% CI: 1.30, 4.43) increased the risk of any type of diarrhea or chronic diarrhea compared to the group which was negative for both infections.

Among HIV infected individuals, any species of intestinal parasite was not associated with diarrhea status. However, *E. histolytica/dispar *was more likely detected among HIV negative subjects who reported acute (OR = 3.3; 95% CI 1.5 to 7.1) and chronic diarrhea (OR = 4.0; 95% CI 1.4 to 11.3) compared with those with no diarrhea. Similarly, *G. lamblia *was more frequently detected in those with acute (OR = 4.6; 95% CI 1.1 to 19.4) and chronic diarrhea (OR = 5.9; 95% CI 1.1 to 32.0).

HIV positive patients with CD4 counts less than 200 cells/μL had reported an excess risk of having diarrhea independent of parasitic infection compared with those having 500 cells/μL and above (OR = 2.7; 95% CI 1.24 to 5.91) (table 4).

The rate of parasitic infection was increased with decreasing CD4 T-cell count among HIV infected individuals (Table 5). The highest infection rate was at CD4 counts of less than 200 cells/μL and it was about six-fold higher compared with individuals having counts greater than 500 cells/μL (OR = 6.3; 95% CI 2.6 to 15.1). Similarly, an increased rate of mixed parasitic infection was observed at the same lower counts of CD4 T-cells (OR = 3.1; 95% CI 1.1 to 8.9). Higher rate of *S. stercoralis *(OR = 3.2; 95% CI 1.4 to 7.3), *I. belli *(OR = 7.5; 95% CI 3.1 to 18.7) and *Cryptosporidium *species (OR = 12.1; 95% CI 5.5 to 26.3) were detected among individuals with CD4 counts less than 200 cells/μL compared with those having 200 cells/μL and above.

**Table 5:** Distribution of HIV-associated parasites in different categories of CD4 T-cell counts, Hawassa Referral Hospital, Ethiopia, 2008

CD4 T-cell count/μL	Total (%) examined	Positive (%) for any parasite	*S. stercoralis*	*I. belli*	*Cryptosporidium *species
< 200	61 (28.5)	51(83.6)	14 (23.0)	18 (29.5)	31(50.8)
200-349	58 (27.1)	35 (60.3)	4 (6.9)	7 (12.1)	7 (12.1)
350-499	46 (21.5)	20 (43.5)	3 (6.5)	1 (2.2)	4 (8.7)
≥ 500	49 (22.9)	22 (44.9)	6(12.2)	0	1(2.0)

## Discussion

This study determined the prevalence and pattern of intestinal parasites among HIV positive and negative individuals to asses if trend of occurrence was evident. The study also attempted to investigate whether the distribution of parasites was affected by immune status, and finally to provide information regarding diarrhea associated parasites. The overall prevalence of intestinal parasites was 55% among the study subjects. Compared to the current study, a lower rate of parasitic infection (39.8%) was shown among individuals with and without HIV/AIDS in south western Ethiopia (Jimma) [14]. Higher rate of parasitic infection among farmers in the present study may be explained by increased occupational exposure to contaminated soil. The preponderance of parasitic infection among merchants may also be due to the common habit of placing fingers in their mouth in attempt to ease counting of money with moisten fingers.

In this study, HIV status was associated with infection of *Cryptosporidium*, *I. belli*, and *S. stercoralis*. However, HIV had non-significant effect to modify prevalence of other protozoan or helminthic infections. In agreement, several other studies reported higher rate of *Cryptosporidium *[10,11,14-16] and *I. belli *infection [10,11] among HIV positive individuals. Predominance of *S. stercoralis *infection in the same sero-group was also reported elsewhere [14,17]. In contrast to our study, however, predominance of *S. mansoni, T. trichiura *and hookworm infection was additionally detected among HIV positive individuals in Jimma [14]. On the other hand, higher rate of *T. trichiura, A. lumbricoides*, and *G. lamblia *in Honduras [16] and *A. lumbricoides, I. butschlii *and *C. mesnili *in Zambia [11] were reported among HIV negative individuals. These reports did not confirm similar trend of parasite occurrence in relation to HIV status.

In agreement with previous studies [10,18] those parasites associated with HIV were more likely encountered as the CD4 T-cell count fell below 200 cells/μL. This may be because immunodeficent patients were either more susceptible to acquire particular parasites and/or unable to clear once infection is established. However, the mechanism by which immunodeficiency facilitate selective establishment of certain parasites is not yet clear. Indeed, the magnitude of impairment on innate or acquired immunity alters the range of pathogens to which the host is susceptible [19].

Mixed infection is a common phenomenon in areas where various types of intestinal parasites are encountered [12]. Although most participants in the present study harbor mixed infection, difference was observed by HIV status. Increased rate of mixed infections among HIV positive individuals, particularly in those with CD4 counts below 200 cells/μL may be because of higher prevalence of certain parasites among the risk group, which favors the frequent mixing up.

It has been reported that diarrhea is an important clinical problem among HIV-infected patients and associated with significant impairments in health-related quality of life [20]. The present study shows that diarrhea is a concern among the participants regardless of their HIV status though it more likely takes chronic course among HIV-infected participants than HIV uninfected group. HIV and various parasitic infections were reported to associate with diarrhea [10,11]. Similarly, in this study, the role of either HIV or parasitic infection independently or as a co-infection to cause any type of diarrhea or chronic diarrhea was significant. A more than twofold increase of diarrhea among patients with CD4 counts less than 200 cells/μL in the present study may re-affirm the view that diarrhea to be an AIDS defining condition [21].

This hospital based cross-sectional study provided preliminary data for further detailed information in the particular area. A study using a longitudinal design with stronger power and representative sample reliably investigates the possible immunologic and epidemiologic interaction between HIV infection and intestinal parasites. Our reliance on participants report to assess symptom and duration of diarrhea may introduce bias. Thus, results of this study should be interpreted in light of the study limitations.

## Conclusion

The high prevalence of intestinal parasitic infection in the study population warrants the urgent need of intervention so as to avoid its consequences. Infection of *Cryptosporidium*, *I. belli *and *S. stercoralis *was significantly higher among HIV positive subjects, particularly in those with lower CD4 T-cell counts. As no cure is available for cryptosporidiosis, people with HIV should be advised on how to avoid infection, including the potential benefits of drinking boiled water and avoiding contact with animals. Screening of HIV infected individuals is also essential for early treatment of *I. belli *and *S. stercoralis *infection. Moreover, raising immune status of HIV infected patients with anti-retroviral therapy may help to reduce acquisition and/or proliferation of HIV associated parasitic infections and the likelihood of experiencing diarrhea.

## Competing interests

The authors declare that they have no competing interests.

## Authors' contributions

SA was the principal investigator for the study; SA, BE, GM and ZA contributed to the design of the study; SA carried out the laboratory works; TS and BE supervised data collection; TS and GM performed the statistical analyses; SA and TS interpreted the result; all authors contributed to the write up. All authors read and approved the final manuscript.

## Pre-publication history

The pre-publication history for this paper can be accessed here:

http://www.biomedcentral.com/1471-2334/9/155/prepub
